# Sliding intercalated autograft for tendon defect reconstruction – The elongation rail technique (ERT)

**DOI:** 10.1016/j.jpra.2025.06.007

**Published:** 2025-06-16

**Authors:** P. Givissis, B. Chalidis, E. Karagergou, N.P. Sachinis, A. Givissis, L. Harhaus

**Affiliations:** a 1st Orthopaedic Department, Aristotle University of Thessaloniki, Papanikolaou Hospital, G. Papanikolaou Avenue, Thessaloniki, Greece; b School of Medicine, European University of Cyprus, Diogenous 6, Nicosia, Cyprus; c BG Klinikum Unfallkrankenhaus, Klinik für Hand-, Replantations- und Mikrochirurgie Charité Universitätsmedizin Berlin, Warener street 7, Berlin, Germany

**Keywords:** Tendon injuries, Tendon rupture, Tendon defect, Tendon repair, Tendon autograft

## Abstract

Tendon defect reconstruction is a challenging procedure that can be complicated by tendon retraction or tendon gap increase after debridement of interposed scar tissue. Although application of tendon autografts from dispensable tendons is considered the gold standard for bridging the gap, donor-site morbidity cannot be ignored. We use a local sliding tendon autograft which can avoid distant graft harvesting and it is adaptable across all tendon zones for reconstruction of tendon defects. A reverse l-type longitudinal incision of the same length with the measured final gap is made at the midsubstance of the proximal tendon stump and the created intercalary tendon segment is transferred distally to bridge the gap, providing an “elongated” proximal stump of the required length. The Elongation Rail Technique has been applied successfully in four patients with flexor tendon defects with favorable results and functional outcome at 6 months follow-up according to Strickland and Glogovac criteria.

## Introduction

Tendon defect can be complicated by tendon retraction, loss of tendon sheath and scarring, interposed pseudotendon formation, or increase of tendon gap after thorough debridement of the frayed tendon ends.^1^, ^2^, ^3^ Reconstruction is challenging, and the presence of open fractures, soft tissue injuries, or delayed surgical intervention further increases the risk of adhesion formation and joint contractures, resulting in suboptimal outcomes.^4^

Reconstruction of the tendinous gap can be addressed with mobilization of the affected tendon and end-to-end repair (if possible), tendon transfer or reconstruction as one- or two-stage procedure, depending on the integrity of the pulley system.^1^^,^^2^^,^^5^ Surgical procedures for reconstruction of tendon defects include the step cut lengthening technique,^2^ the interposition tendon graft technique using mainly the palmaris longus graft,^1^ and the turnover lengthening technique.^3^ However, the above methods have inherent drawbacks related to the length of the tendon gap, the donor site morbidity of graft harvesting, the possible absence of palmaris longus and the creation of a bulky tendon turnover pedicle, respectively.

We report the use of the Elongation Rail Technique (ERT) where a tendon autograft is harvested from the proximal tendon stump, in a length equal to that of the defect. Thus, we overcome the limitations of the aforementioned techniques.

## Case reports

**Figure 1:**
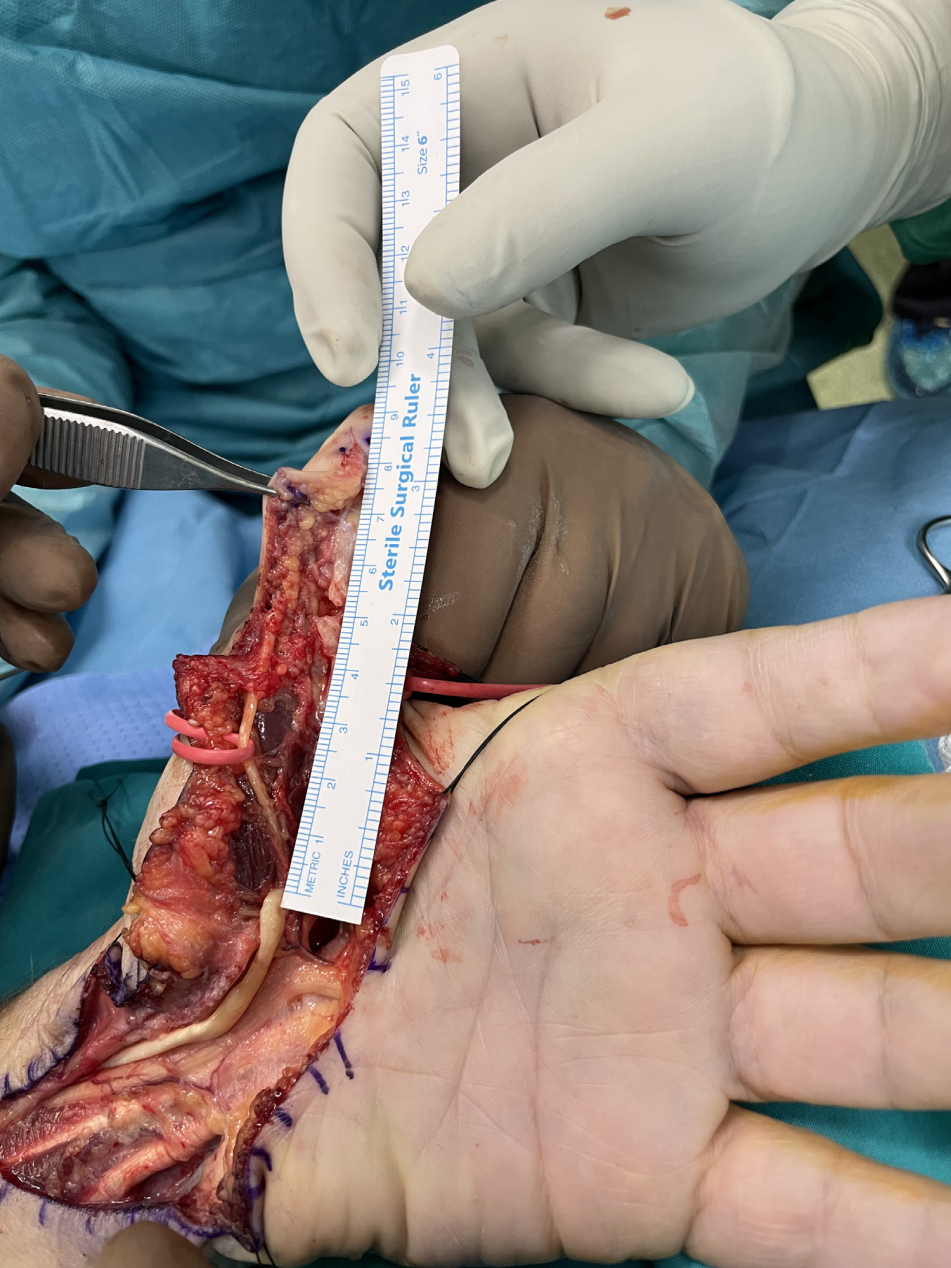
Rupture of FPL tendon in zone 1. Measuring the distance of FPL tendon gap, following retrieval of the proximal stump from its tendon sheath.

**Figure 2:**
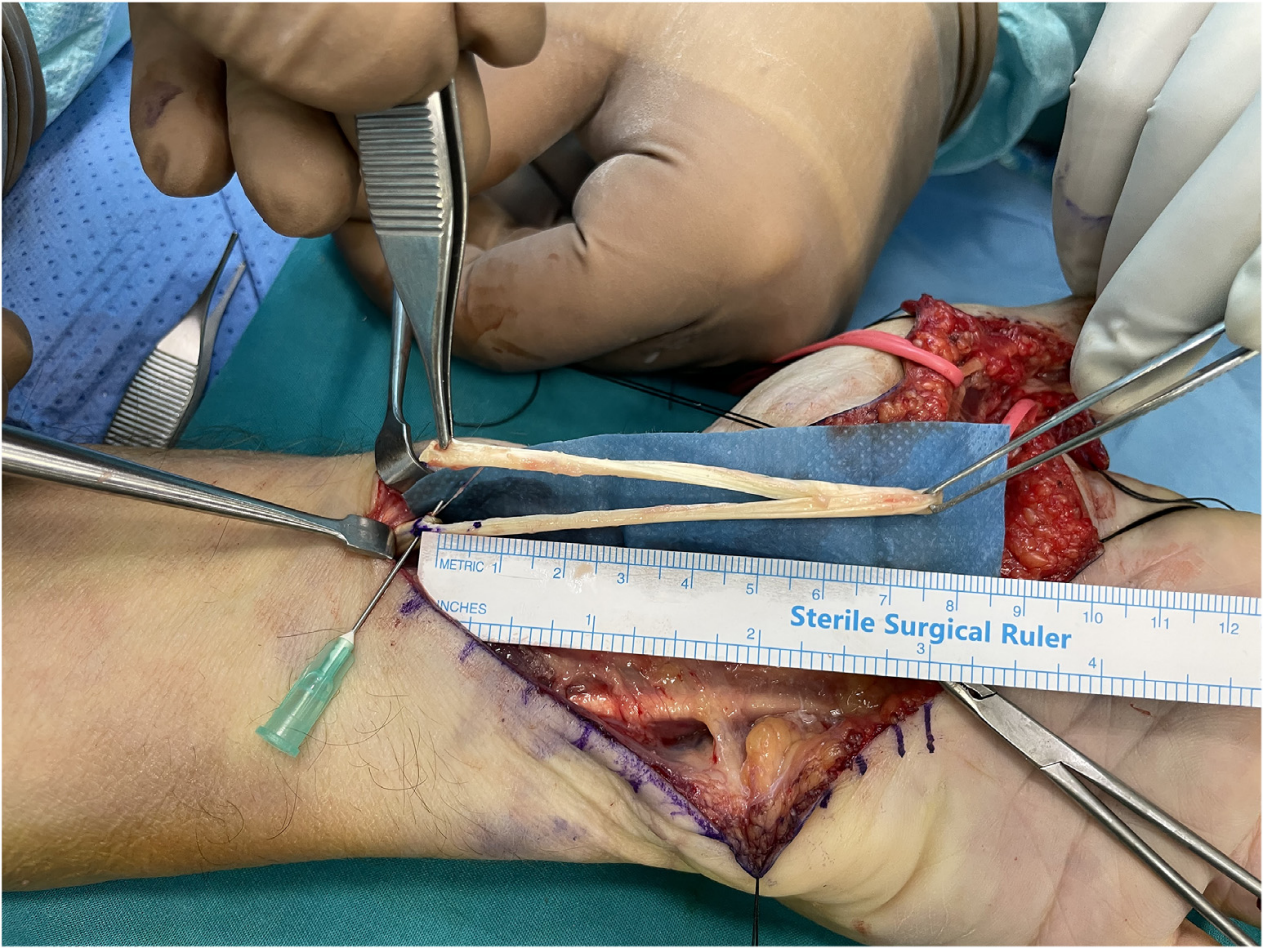
Creation of the tendon autograft by longitudinally splitting half of the proximal stump in a length identical to the measured gap.

**Figure 3:**
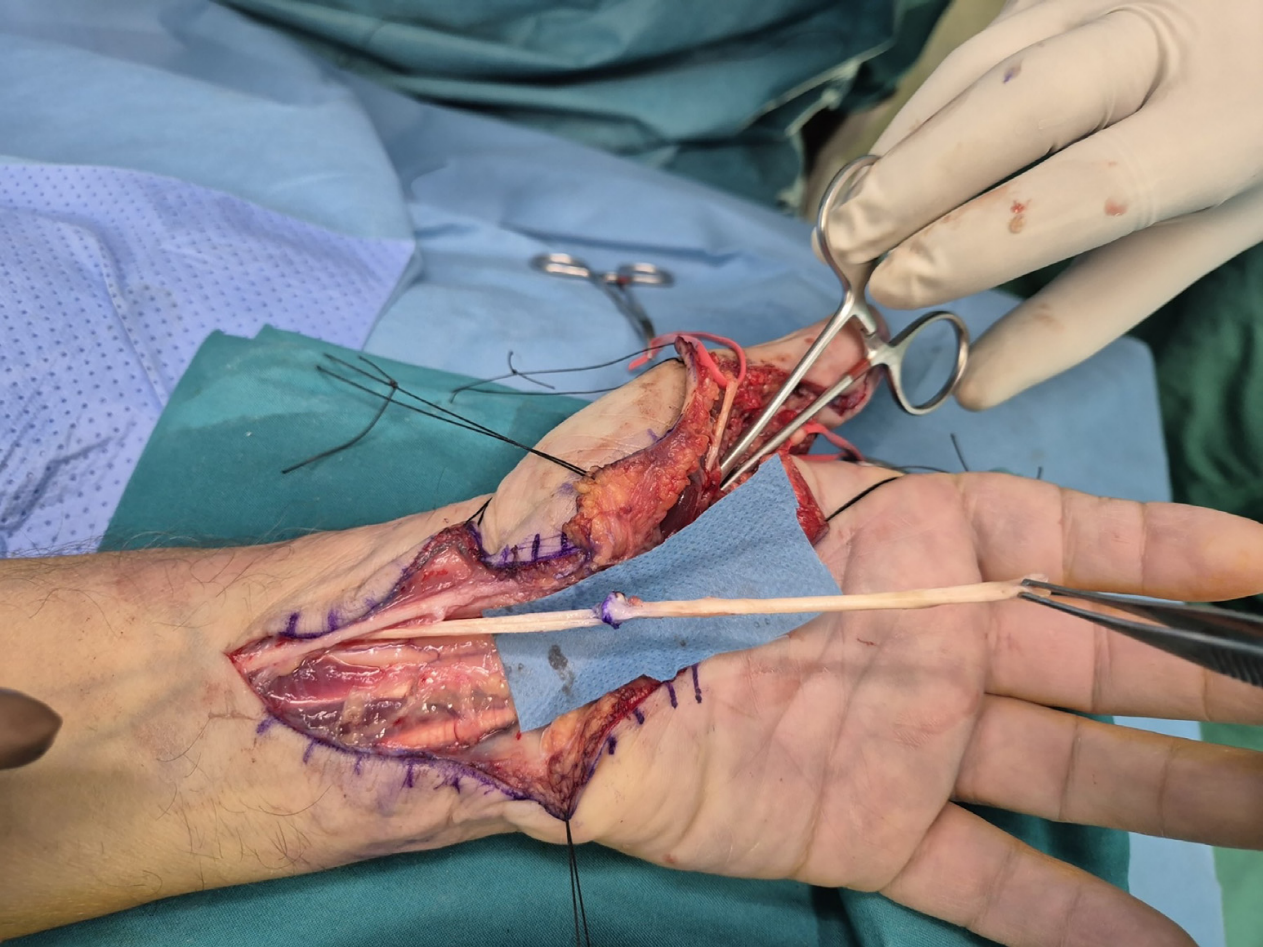
The tendon autograft has been sutured to the proximal tendon stump.

We treated four patients using the ERT technique for tendon rupture of flexor pollicis longus (FPL) (*n* = 3) and flexor digitorum profundus (FDP) (*n* = 1). All patients presented late and the mean time from injury to surgery was 10.5 weeks (range from 7 to 13 weeks). In all patients tendon reconstruction was achieved in one stage. During surgery, removal of fibrotic scarred tissue and debridement of the tendon stumps were performed. The major annular pulleys A2 and A4 were preserved to avoid bowstringing of the tendons. The mean length of tendon gap was 4.4 cm (range from 3.2 to 5.3 cm) and this distance defined the desired length of tendon graft (Figure 1). A reverse l-shaped longitudinal incision, matching the measured gap length, was created at the midpoint of the proximal tendon stump (Figure 2). In cases of FDP reconstruction, where the defect was distal to the lumbrical muscle origin, tendon splitting and harvesting were performed on the ulnar half of the tendon, avoiding to jeopardize the lumbrical muscle origin. The created proximal tendon segment was transferred towards the distal stump as an intercalated segment to bridge the tendon gap, and it was sutured to both proximal and distal stumps in an end-to-end fashion via a 4-strand modified Kessler repair and a running epitendinous suture (Figure 3). The distal juncture was created first and the appropriate graft length was determined in the palm, at the proximal juncture, by evaluating the finger cascade during tenodesis effect. Postoperatively, a dorsal short arm splint in the intrinsic plus position was applied for 6 weeks and the modified Belfast regime for flexor tendon repairs was instructed and supervised by hand therapists.^6^

All patients had an uneventful postoperative period and were followed up for six months. At their last follow-up visit, they reported satisfactory function and could perform all daily living activities without discomfort or pain. The grip and pinch strength and the total active range of movement (AROM) as calculated by the modified Strickland formula^7^ are shown in Table 1.

**Table 1:** Patient demographics and outcome measures.

No	Age	Gender	Ruptured tendon	Time from injury to surgery	Tendon gap (cm)	Grip strength (kg)	Pinch strength (kg)	Total AROM
1	35	male	FDP IF	13 weeks	3.2	45	9.5	74 %
2	62	female	FPL	10 weeks	5	22	4.5	89 %
3	72	female	FPL	7 weeks	4.1	20	5	85 %
4	65	male	FPL	12 weeks	5.3	39	7	93 %

## Discussion

The Elongation Rail Technique where a tendon autograft is harvested from the proximal tendon stump, is a valuable technique for late flexor tendon repair. It can be performed as one stage reconstruction, it allows early active motion therapy and results in satisfactory function. Additionally, it has certain advantages when compared to other tendon reconstruction techniques, such as interposition grafting by using mainly the palmaris longus,^1^ the step cut lengthening,^2^ and the turnover lengthening technique,^3^ because it avoids donor site morbidity and is adaptable to various zones. Contraindications include infection, poor local tissue quality, or insufficient remaining tendon length due to long defects.

Ιnterposition grafting, which is the gold standard for tendon reconstruction, uses palmaris longus, flexor digitorum superficialis of the ring finger or extensor indicis proprius tendon.^8^ In a recent study, an interposition graft was applied in 14 patients with FPL segmental defect, and the authors reported only one failure at 6 weeks postoperatively. Flexion of IP joint and opposition were reduced compared to the contralateral thumb but grip and key pinch strength were similar between injured and non-injured hand. However, the potential donor site morbidity of graft harvesting and the possibility of an absent or hypoplastic palmaris longus are considered inherent drawbacks of this technique.^8^ The main advantage of ERT is that no additional donor tendon is required and the proximal tendon stump supplies the autograft for defect reconstruction.

The step cut tendon lengthening technique was introduced for the segmental reconstruction of FPL defect without the need to sacrifice a donor tendon.^2^ Apart from tendon lengthening, stretching of the FPL muscle along with inter-phalangeal (IP) joint flexion were also applied intraoperatively to manage the closure of the residual tendon gap. The two reported cases were associated with good results in terms of strength and thumb IP joint motion. However, the technique has a narrow zone of application as it could not be applied in segmental tendon loss of >2 cm.^2^ The sliding intercalated tendon autograft can be used alternatively to bridge longer defects in almost all hand zones for flexor or extensor tendon defects, regardless the extent of the tendon gap. It is based on the biomechanical knowledge that preservation of 50 % of the circumference of a flexor tendon is adequate for strength and function. A 4-strand end-to-end tendon repair is advised proximally and distally, especially when within zone 2. Alternatively, a side-to-side or weave technique can be performed at the proximal juncture in the palm to improve the ultimate strength of the construct.

The turnover lengthening technique, which is based on the same principle of harvesting half of the tendon, includes tubularization of the sliding graft which is turned over a pivot point 0.5 to 1 cm from the end of proximal stump.^3^ It has been used in two cases with a 2 cm flexor tendon (FPL) defect and a 1.5 cm extensor tendon (EDC) defect.^3^ Both patients reported a good postoperative outcome and returned to daily activities without significant limitations. The main drawback of this technique is the creation of a bulky tendon turnover pedicle, which would be difficult to apply in tight hand compartments such as the flexor zone II and the extensor zone VII.^3^ This bulky turnover point can be avoided with the ERT technique, as the graft is completely detached and advanced distally to bridge the defect.

In conclusion, the sliding intercalated tendon autograft is an advantageous technique for tendon defect reconstruction and can be applied across all tendon zones. It can avoid distant graft harvesting and donor-site morbidity and it is also useful in cases where the palmaris longus is absent. The satisfactory outcomes observed in the four cases support further evaluation of the ERT technique as a viable reconstructive option for tendon defects. More studies are required to compare the ERT technique with others in prospective and randomized trials.

## Funding

None.

## Ethical consideration

Informed consent was obtained from all individual participants included in the study.

## Conflicts of interest

None.
